# Editorial: Dissecting malaria protective immunity: acquired by natural infection and/or vaccination

**DOI:** 10.3389/fimmu.2026.1830558

**Published:** 2026-04-10

**Authors:** Bernard N. Kanoi, Wang Nguitragool, Nirianne Marie Q. Palacpac, Takafumi Tsuboi

**Affiliations:** 1 Centre for Malaria Elimination, Institute of Tropical Medicine, Mount Kenya University, Thika, Kenya; 2 Mahidol Vivax Research Unit, Faculty of Tropical Medicine, Mahidol University, Bangkok, Thailand; 3 Department of Molecular Tropical Medicine and Genetics, Faculty of Tropical Medicine, Mahidol University, Bangkok, Thailand; 4 Laboratory of Malaria Vaccine Development, Research Institute for Microbial Diseases, The University of Osaka, Suita, Osaka, Japan; 5 Division of Cell-Free Sciences, Proteo-Science Center, Ehime University, Matsuyama, Japan

**Keywords:** malaria, naturally acquired, *Plasmodium*, protective immunity, vaccine

## Introduction

Malaria remains a major global health challenge. Although the WHO has recommended both RTS,S/AS01 (1) and R21/Matrix-M (2) vaccines, their protection is modest and short-lived, reflecting gaps in our understanding of the mechanisms that drive durable immunity. Next-generation malaria vaccines will need to provide stronger, longer-lasting protection, target multiple parasite stages, remain scalable and affordable, and complement existing control strategies. This Research Topic provides a platform to highlight studies on protective immunity against *P. falciparum* and *P. vivax* malaria acquired through natural infection or vaccination. The contributions collectively address three interrelated questions: (i) what immune mechanisms are associated with protection; (ii) how exposure history, age, and physiological state shape immune regulation; and (iii) how these insights can be leveraged to design next-generation malaria vaccines.

## Functional immunity and correlates of protection

Antibodies targeting *P. falciparum* proteins are important for malaria immunity by blocking invasion of liver and red blood cells, preventing sequestration of infected erythrocytes (IEs), facilitating IEs clearance, and reducing transmission (3). The mere presence of antibodies is insufficient for protection. Instead, clinical outcomes depend on their functional properties and underlying cellular mechanisms. In severe malaria, antibodies against PfEMP1 are central to protection. Banda et al. reviewed the critical role of antibodies against PfEMP1, specifically targeting endothelial protein C receptor-binding CIDRα1 domains. These antibodies can prevent microvascular sequestration that drives cerebral malaria, underscoring Fc-mediated effector functions, such as antibody-dependent cellular cytotoxicity and opsonic phagocytosis, in mitigating disease outcomes.

In a Phase IIb controlled human malaria infection trial, Kibwana et al. showed that R21/Matrix-M vaccination induced robust IgG1 and IgG3 responses alongside IgA and IgM. These antibodies demonstrate significant C1q-binding activity and sporozoite inhibition, which are critical for preventing hepatocyte invasion. At the cellular level, the study found an expansion of circulating T follicular helper cells and dominant Th2 and Th17 subsets linked to the maintenance of antigen-specific memory B cells, suggesting that R21/Matrix-M promotes T-dependent responses that help overcome the weak immune memory associated with the NANP repeat region. Complementing this, Bundi et al. established that the functional quality, measured by inhibition of sporozoite invasion and C1q fixation, is a more precise indicator of protection than total IgG levels alone. Overall, functional responses were positively associated with NANP vaccine-induced antibodies.

## Immune regulation across exposure gradients

Immune regulation is a key determinant of how different populations respond to both infection and immunization. Bundi et al. reported a “hypo-responsiveness” phenomenon whereby adults, despite having higher baseline malaria antibodies from prior exposure, generated significantly lower vaccine-induced antibodies and functional activity compared to children and infants. This differential responsiveness is potentially driven by immune tolerance or exhaustion resulting from chronic, life-long exposure to *P. falciparum*.

In the context of pregnancy, Mwai et al. screened 698 recombinant *P. falciparum* antigens and found that antibody breadth increases with gravidity. They identified SURFIN_13.1, SURFIN_4.2, and PfRON3 as being significantly associated with multigravidity, suggesting these targets contribute to parity-dependent protection against placental sequestration. This work challenges the long-standing VAR2CSA-centric paradigm and opens the field to broader antigen prioritization for pregnancy-specific vaccines. Regulatory profiles are also markedly altered in specialized biological niches. Owino et al. investigated the impact of placental malaria (PM) on maternal immune cells and found that infection is associated with downregulation of the transcription factor STAT-6 and the angiogenic factor ANG-1 in decidual macrophages. Because decidual macrophages are essential for maintaining maternal-fetal tolerance and supporting a competent vascular network, this PM-induced dysregulation likely impairs placental function and contributes to adverse birth outcomes.

## Age, antigen specificity, and the architecture of natural immunity

At a more granular resolution of how humoral immunity evolves across the human lifespan, Yuguchi et al. conducted a comprehensive profiling of antibody responses to 1,307 *P. falciparum* proteins in Burkinabe children. They identified 173 candidate protein targets associated with reduced risk of clinical malaria and showed that the greatest expansion in the breadth of the humoral repertoire occurs during the first two years of life, after waning of maternal IgG. These findings provide compelling evidence that early-life immunity is neither passive nor simplistic but is instead actively shaped by exposure history, antigenic diversity, and immune maturation.

The maturation of antibody quality likewise follows age-dependent patterns. Lugaajju et al. used surface plasmon resonance to show that antibody affinity for two merozoite antigens, AMA1 and MSP2, matures gradually over the first nine months of life. In contrast, affinity for CSP remained low and stable throughout the study period, suggesting that structural characteristics of CSP may inherently limit the induction of high-affinity antibody responses during natural infection. This decoupling of quantity and quality underscores the limitations of antibody titre-based correlates of protection and highlights affinity maturation as a critical, antigen-specific bottleneck in the development of natural immunity.

In the case of *P. vivax*, Soares et al. found that naturally acquired anti-PvRipr IgG and IgM responses were detected in individuals exposed to malaria in the Brazilian Amazon. The findings are noteworthy given that the orthologous Ripr antigen in *P. falciparum* is currently in preclinical (4, 5) and clinical phases (ClinicalTrials.gov: NCT07183371).

Structural determinants also play a role in the immune system’s recognition of polymorphic antigens. Zerebinski et al. demonstrated that naturally acquired IgG responses against MSP2 primarily target the highly polymorphic variable regions rather than the conserved termini. Alphafold3 modeling predicted extensive structural heterogeneity and epitope masking in the conserved termini upon antigen oligomerization, which likely serves as a parasite evasion mechanism.

Mulamba et al. found that naturally acquired antibodies to transmission-blocking targets Pfs230D1M and Pfs48/45 are prevalent, stable, and age-associated in Tanzanian populations. Transmission-reducing activity was detectable in individuals who were highly reactive to Pfs230D1M and Pfs48/45, indicating that antibody responses were functional. These findings suggest that naturally primed populations may be well suited for boosting transmission-blocking immunity, with direct implications for vaccine deployment strategies.

## Vaccine-induced immunity: from platforms to mechanisms

To overcome the limitations of current vaccines, research is also shifting toward multi-stage antigens and optimized adjuvant formulations. Plieskatt et al. reported the clinical evaluation of ProC6C, a novel multi-stage subunit vaccine candidate encompassing sequences from PfCSP, Pfs48/45, and Pfs230. In a Phase 1 trial in Burkina Faso, ProC6C adjuvanted with Matrix-M elicited a bi-functional antibody response that inhibits both sporozoite invasion of hepatocytes and parasite development in the mosquito midgut. This study demonstrated that low-valency repeat sequences could induce functional PfCSP antibodies comparable to those induced by high-valency platforms such as RTS,S/AS01, potentially avoiding limitations in B-cell affinity maturation.

Adjuvant studies remain a critical frontier for optimizing vaccine formulations. Bundi et al. found that increasing the Matrix-M adjuvant dose (from 25µg to 50µg) in infants significantly enhanced the functional quality of the response, resulting in higher C1q fixation and sporozoite inhibition without necessarily increasing antibody magnitude. Tandel et al. also explored the dual TLR agonist combination adjuvant, Poly(I:C) and R848 for liver-stage malaria antigens. The observed antibody responses, in terms of functionality and durability, illustrate the challenges in demonstrating the role of adjuvants in modulating the quality, as well as quantity, of the immune response.

The need for strain-transcending interventions has also led to the exploration of broadly inhibitory monoclonal antibodies. Banda et al. highlighted C7 and C74 monoclonal antibodies targeting the CIDRα1 domain of PfEMP1 as promising therapeutic avenues for preventing parasite sequestration associated with cerebral malaria. Finally, Jun et al. emphasized that future PfRh5-based vaccines must integrate geographic antigenic variation and emerging mutations to ensure effectiveness against genetically diverse parasites circulating in endemic regions.

## Converging insights and future directions

This Research Topic collectively demonstrates that durable malaria protection stems from coordinated humoral, cellular, and regulatory immune networks rather than antibody magnitude alone. Next-generation vaccines will require multi-stage, multivalent designs and strategies that enhance antibody quality/long-term memory, and systems-level integration to achieve sustainable, population-wide protection.
